# Dependence and clinical fragility in hematopoietic stem cell transplant and CAR-T therapy: a retrospective study

**DOI:** 10.1007/s00520-025-09925-5

**Published:** 2025-09-26

**Authors:** Marco Cioce, Matteo Raponi, Chiara Visintini, Domenico Pascucci, Stefano Botti, Patrizia Laurenti, Carmen Nuzzo, Giuseppe Vetrugno, Nicola Nicolotti, Angela Iula, Simona Sica, Patrizia Cornacchione, Simona Calza, Sarah Jayne Liptrott

**Affiliations:** 1 Direction of Health Professionals, Fondazione Policlinico Universitario A. Gemelli IRCCS, Largo A. Gemelli, 8, Rome, Italy; 2 Department of Women, Child and Public Health Sciences, Fondazione Policlinico Universitario A. Gemelli IRCCS, Rome, Italy; 3 Division of Hematology and Stem Cell Transplantation, S. Maria della Misericordia University Hospital, Azienda Sanitaria Universitaria Friuli Centrale, Udine, Italy; 4 Health Management, Fondazione Policlinico Universitario A. Gemelli IRCCS, Rome, Italy; 5 Department of Life Sciences and Public Health, Università Cattolica del Sacro Cuore, Rome, Italy; 6 Azienda USL-IRCCS Di Reggio Emilia, Hematology Unit, Reggio Emilia, Italy; 7 Department of Health Surveillance and Bioethics, Section of Legal Medicine, Università Cattolica del Sacro Cuore, Fondazione Policlinico Universitario A. Gemelli IRCCS, Rome, Italy; 8 Quality and Accreditation Unit, Fondazione Policlinico Universitario A. Gemelli IRCCS, Rome, Italy; 9 Dipartimento Di Scienze Di Laboratorio Ed Ematologiche, Fondazione Policlinico Universitario A. Gemelli IRCCS, Rome, Italy; 10 Sezione Di Ematologia, Dipartimento Di Scienze Radiologiche Ed Ematologiche, Università Cattolica del Sacro Cuore, Rome, Italy; 11 Dipartimento Diagnostica Per Immagini E Radioterapia Oncologica, Fondazione Policlinico Universitario A. Gemelli IRCCS, Rome, Italy; 12 IRCCS Istituto Giannina Gaslini, Direction of Health Professions, Genoa, Italy; 13 Oncology Institute of Southern Switzerland, Ente Ospedaliero Cantonale (EOC), Nursing Development and Research Unit, Bellinzona, Switzerland; 14 Regional Hospital of Bellinzona E Valli, Ente Ospedaliero Cantonale (EOC), Department of Nursing, Bellinzona, Switzerland

**Keywords:** Car-T therapy, Hematopoietic stem cell transplantation, Dependence, Fragility, Complication

## Abstract

**Purpose:**

Autologous and allogeneic hematopoietic stem cell transplantation and CAR-T cell therapy are standard treatments for patients affected by hematologic malignancies at different stages of their clinical pathway. Several adverse events accompany these therapies during patients’ hospital stay, including psycho-emotional distress and various functional and social impairments.

**Methods:**

In this monocentric study on 498 patients, various clinical and social fragility indices, registered within clinical documentation during the hospital stay, specifically at admission (within 24 h of entry) and discharge (less than 24 h prior to leaving), were retrospectively assessed with the aim of describing their trends and evaluating for differences between type of cell therapy and underlying disease.

**Results:**

Functional independence mean score was lower in allogeneic patients at admission (*p* = 0.001) and at discharge (*p* = 0.001), while a higher risk of falling at discharge (*p* = 0.001), an increased risk of clinical deterioration at admission (*p* = 0.001) and at discharge (*p* = 0.011), and increased complexity of care at both time points (*p* = 0.001) were observed in patients undergoing CAR-T compared to patients undergoing allogeneic or autologous HSCT procedures. Acute lymphoid leukemia patients had significantly worse mean scores at both time points for functional independence, risk of falling, clinical deterioration, care complexity, level of pain (*p* =  < 0.001); while acute myeloid leukemia patients had higher risk of skin pressure injuries at discharge (*p* = 0.036).

**Conclusion:**

Our findings may contribute to improving personalized assessment strategies in the field of cell therapy, including both CAR-T therapy and stem cell transplantation. They highlight the increased risk and complexity of care, particularly in patients receiving CAR-T therapy, while emphasizing the need for tailored management approaches across different cell therapy modalities.

## Background

Hematological malignancies represent challenging clinical conditions, characterized by high levels of morbidity and mortality, especially in patients with multiple refractory diseases. These diseases account for 1.2 million cases, approximately 7% of newly diagnosed cancer cases worldwide each year [1]. Among them, leukemia, lymphoma, and multiple myeloma (MM) represent a significant proportion. Chemotherapy and hematopoietic stem cell transplant (HSCT), as traditional and common treatments for these conditions, are gradually being complemented by novel therapies, such as chimeric antigen receptor-modified cell therapy (CAR-T) [2].

Complications of HSCT can be categorized based on time of manifestation into two groups: those occurring during the pre-engraftment period (from the start of the conditioning regimen to neutrophil recovery) and the early post-engraftment period. Complications in the pre-engraftment period are typically a result of the toxicities of the conditioning regimen, leading to issues such as pancytopenia, gastrointestinal toxicities, infections, and organ dysfunction in the recipient [3]. In the early post-engraftment period, acute graft-versus-host disease (aGVHD) may present, which is exclusively seen in allogeneic transplantation and occurs when transplanted immune cells recognize the recipient as foreign and mount an immune reaction. It usually affects the skin, gastrointestinal system, and liver, with common manifestations including rash, watery diarrhea, persistent nausea or vomiting, anorexia, cholestatic jaundice, and abnormalities in liver function tests. During this period, patients also remain at risk of infectious complications [4].

The application of chimeric antigen receptor (CAR) T-cell therapy is associated with adverse reactions, including cytokine release syndrome (CRS), immune effector cell-associated neurotoxicity syndrome (ICANS), off-target effects, anaphylaxis, infections associated with CAR-T-cell infusion (CTI), tumor lysis syndrome (TLS), B-cell dysplasia, hemophagocytic lymphohistiocytosis (HLH)/macrophage activation syndrome (MAS), and coagulation disorders [5–7]. A common feature among the three different types of treatment (autologous HSCT, AUTO HSCT; allogenic HSCT, ALLO HSCT; and CAR-T cell therapy) is the high levels of psycho-emotional distress they generate [8–11].

A comprehensive patient assessment is crucial to evaluate for the presence of risks associated with common complications. This assessment should address physical aspects (e.g., patient autonomy), neurocognitive factors, biometabolic considerations, hemodynamic aspects, and social factors. Dependence and Clinical-Social Fragility instruments (DEP-CSF indices) are commonly used to assess a series of risks to which patients may be susceptible in different care settings [12]. The DEP-CSF indices permit evaluations of different activities and risk factors such as care complexity (Index of Caring Complexity—ICC) [13], functional dependence in activities of daily living (Barthel Index) [14], pressure injury risk (Braden Scale) [15], risk of clinical deterioration (Modified Early Warning Score—MEWS) [16], fall risk (Conley Scale) [17], pain (Numerical Rating Scale—NRS) [18], need of a more comprehensive discharge plan (Blaylock Risk Assessment Screening Score—BRASS) [19, 20], and alteration in cognitive status (cognitive/perceptive functional model, for mental conditions and for behavior) [21].

Some of these DEP-CSF indices or their components are commonly used to assess patients undergoing HSCT or CAR-T therapy during out-patient follow-up visits; some studies report how they are employed to monitor symptoms through patient-reported outcomes (PROs) [22–24]. This provides an evidence-based approach to detecting symptoms that can offer critical information to clinicians, thereby improving clinical management.

Although there are different DEP-CSF indices based on various risk factors, the current literature lacks studies describing their use in hospitalized adult patients undergoing allogeneic HSCT, autologous HSCT, or CAR-T therapy, and evidence regarding changes that these indices may undergo based on the type of cell therapy received.

The primary objective of this study aims to investigate the relationship between HSCT (autologous and allogeneic) and CAR-T therapy, and scores DEP-CSF indices; the secondary objective was to assess how these indices change in response to the hematological indications for HSCT and CAR-T, as well as to evaluate the relationship between DEP and complications.

## Methods

### Study design

A monocentric retrospective study was conducted at Fondazione Policlinico Universitario A. Gemelli IRCCS (Rome, Italy), by retrieving data of inpatients from the electronic health records. This study was conducted in accordance with the Declaration of Helsinki and was approved by the local Ethics Committee (Comitato Etico Territoriale Lazio Area 3) on April 7, 2022 (Prot. 12720/22, ID: 4859).

### Study population

All patients over the age of 18 were enrolled, who had undergone HSCT (allogeneic and autologous) or had been treated with CAR-T therapy between the 1 of January 2021 and the 30 of June 2023.

### Sample size

As no similar study has been published in the literature, we assumed a dz (effect size) value of 0.5.

The sample size was calculated considering a mixed model ANOVA with repeated measures, with alpha = 0.01 and power 80%, delta = 0.6325 on two groups; and a variance between groups = 0.0200 and a variance “between-within groups” = 0.05 for two or more repeated measures with a correlation index rho = 0.9. Considering possible drop-out, at least 140 patients needed to be recruited.

### Endpoints: DEP-CSFindices

The DEP-CSF indices comprise a series of validated tools used to assess physical, functional, and social vulnerability in hospitalized patients undergoing cell therapy. These include the Barthel Index [14], which evaluates independence in activities of daily living (ADLs), ranging from 0 (complete dependence) to 100 (full independence), and the Braden Scale [15], which assesses the risk of pressure injuries through six subscales such as nutrition and sensory perception. The Conley Scale [17] is used to identify fall risk (cutoff ≥ 2), while the Modified Early Warning Score (MEWS) [16] monitors clinical deterioration based on vital parameters. Pain levels are assessed through the Numerical Rating Scale (NRS) [18], and discharge planning complexity is evaluated using the Blaylock Risk Assessment Screening Score (BRASS) [19, 20], where higher scores indicate a greater need for coordinated post-discharge care. The Index of Caring Complexity (ICC) [13] estimates the level of care intensity based on clinical support needs and patient dependency. Finally, the cognitive–perceptual pattern [21] detects changes in mental and behavioral function and aligns with the cognitive model of professional nursing assessments used in various Italian healthcare settings.

### Clinical outcomes

The clinical outcomes selected in this study were chosen for their specific relevance to the three main cell therapy modalities under investigation. GVHD and VOD are typically associated with allogeneic HSCT, while cytokine release syndrome (CRS) and immune effector cell-associated neurotoxicity syndrome (ICANS) are hallmark complications of CAR-T therapy [5–7]. In contrast, autologous HSCT is generally associated with a lower complication profile and these events are rarely observed. Sepsis and inpatient mortality were included as common, cross-cutting outcomes across all procedures. In this context, “inpatient mortality” refers specifically to deaths occurring during the same hospitalization period for the cell therapy and is therefore distinct from broader survival or long-term mortality outcomes.

The study evaluated several key clinical outcomes and complications associated with HSCT and CAR-T therapies [3–7]. In this context, complications refer to specific treatment-related adverse events occurring during or prior to hospitalization for cell therapy, whereas clinical outcomes encompass broader endpoints such as functional decline, discharge status, or mortality.

The following were considered:

- Acute graft-versus-host disease (aGVHD): The presence and severity of acute GvHD were assessed based on the standardized criteria, which categorize the condition according to the extent of organ involvement and clinical manifestations.
- Venous occlusive disease (VOD): The occurrence of VOD, a potentially severe complication characterized by hepatic sinusoidal obstruction leading to liver dysfunction, was documented according to established diagnostic criteria, considering clinical and laboratory findings.
- Cytokine release syndrome (CRS): This inflammatory response, commonly associated with CAR-T therapy, was evaluated in terms of severity and progression, following established grading systems that account for symptoms such as fever, hypotension, and organ dysfunction.
- Neurologic toxicities: Neurological complications, including immune effector cell-associated neurotoxicity syndrome (ICANS) and other manifestations such as confusion, seizures, or encephalopathy, were monitored and categorized based on severity.
- Sepsis: The incidence of sepsis, a life-threatening systemic infection that can arise as a complication of immunosuppression and treatment-related toxicities, was recorded and classified according to clinical and microbiological findings.
- Mortality: Overall survival and treatment-related mortality were assessed, taking into account both early and late complications, as well as disease progression.

### Statistical analysis

The sample has been described in its socio-demographic and clinical characteristics through descriptive statistical techniques. The qualitative variables were described using absolute frequencies and percentages, while the quantitative variables were synthesized through mean and standard deviation. The one-way ANOVA test was used to determine statistical differences among the means of multiple data groups. To analyze group differences with “nominal” dependent variables, the Chi-square non-parametric statistical test (without assumptions about the distribution) was employed.

Logistic regression analysis was performed to assess the relationships with binary outcomes from DEP-CSF indices, which included Barthel Index [dependent (≤ 75) vs. independent (> 75)], Braden Scale [at risk (≤ 19) vs. not at risk (> 19)], sensory perception subscale of the Braden Scale [at risk (≤ 3) vs. not at risk (> 3)], nutrition subscale of the Braden Scale [at risk (≤ 3) vs. not at risk (> 3)], BRASS [high risk (≥ 11) vs. low risk (< 11)], Conley Scale [at risk (≥ 2) vs. not at risk (< 2)], MEWS [at risk (≥ 2) vs. not at risk (< 2)], and NRS [significant pain (≥ 1) vs. mild/no pain (< 1)].

Independent variables included in the logistic regression were treatment type, indications for HSCT and CAR-T, complications, and DRG costs. Dependent variables were the Dependence and Clinical-Social Fragility Index (DEP-IDX), a composite construct derived from the DEP-CSF indices. The models were adjusted for confounders such as age, sex, baseline condition, and comorbidities. Adjusted odds ratios (ORs) with 95% confidence intervals (CIs) were reported to quantify the associations. The DEP-IDX was operationalized by binarizing each DEP-CSF scale according to clinical cutoffs, allowing for logistic regression analysis.

Data were stored and managed in spreadsheets of Microsoft Excel 2016. Statistical analysis was carried out through Stata [17.1 for Windows (64-bit Intel)]. The statistical significance was set at *p* < 0.05.

## Results

Although admission and discharge assessments were collected consistently for all patients, the length of hospitalization between these time points varied depending on the procedure type and complications. In general, patients undergoing autologous HSCT had shorter stays, while allogeneic HSCT and CAR-T recipients experienced longer hospitalizations, especially in the presence of complications such as aGVHD or CRS. The “other” category under place of discharge includes transfers to rehabilitation or long-term care facilities. “Sepsis” and “sepsis and other” are mutually exclusive; the latter includes cases where sepsis occurred alongside other complications. All complications refer to events occurring during the hospitalization for cell therapy.

### Sample characteristics

The study enrolled 498 participants, predominantly male (59.2%), with a mean age (± SD) of 56 years (61–70 years, 38.8%); the majority of the population was unemployed (59.8%). The main hematological diagnoses on admission were multiple myeloma/plasma cell dyscrasias (32.3%) and lymphoma (29.9%). Most patients were undergoing autologous HSCT (50.4%), followed by allogeneic HSCT (38.8%) and CAR-T therapy in 54 cases (10.8%). The most recurrent post-treatment complications were sepsis (5.6%) and aGVHD (5.2%); the mean duration of hospital stay was between 11 and 20 days (39.2% of cases). The distribution of costs related to diagnosis-related groups (DRGs) showed that 86.2% of cases had costs below €60,000, while 13.8% of cases had costs equal to or above €60,000. Table 1 shows the characteristics of the sample.

**Table 1:** Characteristics of the sample

		***N***	***%***
**Gender**	*Male* *Female*	295 203	**59.2** **40.8**
**Age ranges (yrs)**	*21–30* *31–40* *41–50* *51–60* *61–70* *71* +	23 35 67 156 193 24	**4.6** **7.0** **13.5** **31.3** **38.8** **4.8**
**Nationality**	*Italian* *Not Italian*	468 30	**94.0** **6.0**
**Living local to the hospital**	*Yes* *No*	368 130	**73.9** **26.1**
**Social status**	*House wife/husband* *Unemployed* *Looking for work* *Employed* *Retired* *Student*	37 297 10 136 11 6	**7.4** **59.8** **2** **27.4** **2.2** **1.2**
**Place of discharge**	*Death* *Discharge home* *Other*	17 474 7	**3.4** **95.2** **1.4**
**Diagnosis at admission**	*ALL* *AML* *Ly* *MDS/MPS* *MM/PCD* *Other*	32 79 149 51 161 26	**6.4** **15.9** **29.9** **10.2** **32.3** **5.2**
**Treatment**	*Allogeneic HSCT* *Autologous HSCT* *Car-T therapy*	193 251 54	**38.8** **50.4** **10.8**
**Diagnosis-related group costs**	< *60.000€*	431	**86.2**
	≥ *60.000€*	69	**13.8**
**Hospital stay duration (days)**	*11–20* *21–30* *31–40* *41–50* *51–60* *61* +	195 127 70 65 19 22	**39.2** **25.5** **14.1** **13.1** **3.8** **4.4**
**Complications**	*No* *aGVHD* *VOD* *CRS/Neurologic toxicities* *Sepsis* *Sepsis and other*	398 26 12 25 28 9	**79.9** **5.2** **2.4** **5.0** **5.6** **1.8**

Living locally: residing in the same region as the transplant center; *ALL* acute lymphocytic leukemia, *AML* acute myeloid leukemia, *Ly* lymphoma, *MDS/MPD* myelodysplastic syndromes/myeloproliferative diseases, *MM/PCD* multiple myeloma/plasma-cell disorders, *HSCT* hematopoietic stem cell transplantation, *aGVHD* acute graft-versus-host disease, *VOD* veno-occlusive disease, *CRS* cytokine release syndrome

### Clinical outcomes and fragility indices by procedure type

#### Autologous HSCT (Auto-HSCT)

Autologous HSCT was performed in 50.4% of patients (Table 1), primarily with diagnoses of multiple myeloma/plasma cell dyscrasias (63.3%). These patients were more likely to reside near the hospital, specifically living locally within the same region as the transplant center (80.5%) and had the lowest in-hospital mortality rate (0%) (Table 2). The average hospital stay was shorter than in other groups. Functional independence scores (Barthel Index) were high at both admission and discharge (97.3 and 96.8, respectively), and risk scores for pressure injuries (Braden), falls (Conley), and clinical deterioration (MEWS) were all favorable (Table 4). Auto-HSCT patients also had the lowest incidence of complications such as sepsis and experienced significantly lower care complexity (ICC). They were also less likely to incur high DRG-related costs (only 0.8% ≥ €60,000) (Table 2). Logistic regression confirmed a reduced risk of dependency (OR 0.211; *p* = 0.009) and clinical deterioration (OR 0.110; *p* = 0.034) in this group, as well as better nutritional scores (OR 0.151 at discharge, *p* < 0.001) (Table 5).

**Table 2:** Proportion of patients undergoing each procedure by characteristics of the sample and results of the univariate analysis (Chi-square)

	***Allogeneic HSCT*** ***%***	***Autologous HSCT*** ***%***	***Car-T therapy*** ***%***	***P*. value**
**Gender**				
*Male* *Female*	59.07 40.93	60.16 39.84	55.56 44.44	0.821
**Living locally**				
*Yes* *No*	62.18 37.82	80.48 19.52	85.19 14.81	** < 0.001**
**Nationality**				
*Italian*	94.82	94.42	88.89	0.247
*Not Italian*	5.18	5.58	11.11	
**Social status**				
*House wife/husband*	9.38	5.18	11.11	0.133
*Unemployed*	61.46	58.17	61.11	
*Looking for work*	0	3.59	1.85	
*Employed*	26.56	29.08	22.22	
*Retired*	2.08	1.99	3.70	
*Student*	0.52	1.99	0	
**Place of discharge**				
*Death*	8.29	0	1.85	** < 0.001**
*Discharge home*	91.19	98.01	96.3	
*Other*	0.52	1.99	1.85	
**Diagnosis-related group costs**				
< *60.000€*	66.84	99.20	98.15	** < 0.001**
≥ *60.000€*	33.16	0.80	1.85	
**Diagnosis at the admission**				
*ALL*	14.51	0	7.41	** < 0.001**
*AML*	38.6	1.59	0	
*Ly*	8.81	33.47	88.89	
*MDS/MPS*	26.42	0	0	
*MM/PCD*	0.52	63.35	1.85	
*Other*	10.88	1.59	1.85	
**Complications**				
*No* *aGVHD* *VOD* *CRS/Neurologic toxicities* *Sepsis* *Sepsis and other*	67.3 13.4 6.2 0.0 8.8 4.1	96.8 0.0 0.0 0.0 3.2 0.0	46.3 0.0 0.0 46.3 5.6 1.8	** < 0.001**
**Fall**				
Yes	2.07	1.99	0	0.608
No	97.93	98.01	100	
Mental state on admission*				
Normal	94.71	97.73	88.89	** < 0.001**
Altered	5.29	2.27	11.11	
Mental state at discharge*				
Normal	99.48	97.71	95.56	0.138
Altered	0.52	2.29	4.44	
Space–time disorientation on admission*				
Yes	0.53	1.80	0	0.388
No	99.47	98.20	100	
Space–time disorientation at discharge*				
Yes	3.74	1.79	2.44	0.529
No	96.26	98.21	97.56	
Altered reality perception on admission*				
Yes	0	1.22	0	0.246
No	100	98.78	100	
Altered reality perception at discharge*				
Yes	2.69	1.21	2.44	0.610
No	97.31	98.79	97.56	

Living locally: residing in the same region as the transplant center; *ALL* acute lymphocytic leukemia, *AML* acute myeloid leukemia, *Ly* lymphoma, *MDS/MPD* myelodysplastic syndromes/myeloproliferative diseases, *MM/PCD* multiple myeloma/plasma-cell disorders, *HSCT* hematopoietic stem cell transplantation, *aGVHD* acute graft-versus-host disease, *VOD* veno-occlusive disease, *CRS* cytokine release syndrome

^*^Cognitive–perceptual pattern

#### Allogeneic HSCT (Allo-HSCT)

Allogeneic HSCT was administered to 38.8% of patients (Table 1), with a predominance of acute leukemias: AML (38.6%) and MDS/MPS (26.4%). These patients had the highest in-hospital mortality (8.29%) and more frequently lived farther from the hospital (only 62.2% lived locally) (Table 2). Hospital stays were generally longer, and complication rates were highest, particularly for aGVHD (13.4%), VOD (6.2%), and sepsis (8.8%) (Table 2 and Table 3). Functional independence (Barthel Index) was lowest at both admission and discharge (93.4 and 90.4, respectively). ICC scores indicated high care complexity (Table 4). Allo-HSCT patients were more likely to exceed €60,000 in DRG-related costs (33.2%) (Table 2). These findings highlight the intensive nature and resource demands of allogeneic transplantation.

**Table 3:** Proportion of patients with each pathology by characteristics of the sample and results of the univariate analysis (Chi-square)

	***ALL*** **%**	***AML*** **%**	***Ly*** **%**	***MDS/MPS*** **%**	***MM/PCD*** **%**	***Other*** **%**	***P*. value**
**Gender**							
Male	62.50	50.63	59.73	54.90	61.49	73.08	0.372
Female	37.50	49.37	40.27	45.10	38.51	26.92	
**Living locally**							
*Yes*	59.38	78.48	77.18	43.14	83.85	57.69	** < 0.001**
*No*	40.63	21.52	22.82	56.86	16.15	42.31	
**Nationality**							
*Italian*	93.75	91.14	92.62	100.00	94.41	96.15	0.398
*Not Italian*	6.25	8.86	7.38	0.00	5.59	3.85	
**Social status**							
*House wife/husband*	15.63	11.54	3.36	11.76	6.83	3.85	0.375
*Unemployed*	62.5	57.69	62.42	56.86	57.76	65.38	
*Looking for work*	0	0	2.68	0	3.11	3.85	
*Employed*	18.75	24.36	28.19	29.41	29.19	26.92	
*Retired*	3.13	5.13	0.67	1.96	2.48	0	
*Student*	0	1.28	2.68	0	0.62	0	
**Place of discharge**							
*Death*	3.13	5.06	1.34	9.8	0	19.23	** < 0.001**
*Discharge home*	96.88	94.94	95.97	88.24	98.76	80.77	
*Other*	0	0	2.68	1.96	1.24	0	
**Treatment**							
*Allogeneic HSCT*	87.5	94.94	11.41	100	0.62	80.77	** < 0.001**
*Autologous HSCT*	0	5.06	56.38	0	98.76	15.38	
*CAR-T therapy*	12.5	0	32.21	0	0.62	3.85	
**Diagnosis-related group costs**							
< *60.000€*	68.75	69.62	96.64	56.86	98.76	84.62	** < 0.001**
≥ *60.000€*	31.25	30.38	3.36	43.14	1.24	15.38	
**Complications**							
*No* *aGVHD* *VOD* *CRS/Neurologic toxicities* *Sepsis* *Sepsis and other*	71.9 6.2 15.6 3.2 0.0 3.1	67.1 12.7 8.8 0.00 7.6 3.8	77.8 2.7 0.0 14.8 3.4 1.3	80.4 9.8 0.0 0.0 7.8 2.0	94.4 0.6 0.0 0.6 4.34 0.00	50.0 15.4 0.0 3.8 23.1 7.7	** < 0.001**
**Fall**							
Yes	0	2.53	2.68	1.96	1.24	0	0.670
No	100	97.47	97.32	98.04	98.76	100	
Mental state on admission*							
*Normal*	90.32	98.7	94.74	92	97.39	91.3	0.218
*Altered*	9.68	1.3	5.26	8	2.61	8.7	
Mental state at discharge*							
*Normal*	96.77	100	97.37	100	97.37	100	0.516
*Altered*	3.23	0	2.63	0	2.63	0	
Space–time disorientation on admission*							
*Yes*	0	0	0.96	2.08	1.77	0	0.767
*No*	100	100	99.04	97.92	98.23	100	
Space–time disorientation at discharge*							
*Yes*	3.23	1.32	2.86	8.16	1.77	0	0.225
*No*	96.77	98.68	97.14	91.84	98.23	100	
Altered reality perception on admission*							
*Yes*	0	0	0.98	0	0.89	0	0.890
*No*	100	100	99.02	100	99.11	100	
Altered reality perception at discharge*							
*Yes*	3.33	1.32	1.94	6.12	0.89	0	0.333
*No*	96.67	98.68	98.06	93.88	99.1	100	

Living locally: residing in the same region as the transplant center; *ALL* acute lymphocytic leukemia, *AML* acute myeloid leukemia, *Ly* lymphoma, *MDS/MPD* myelodysplastic syndromes/myeloproliferative diseases, *MM/PCD* multiple myeloma/plasma-cell disorders, *HSCT* hematopoietic stem cell transplantation

^*^Cognitive–perceptual pattern

**Table 4:** Mean scores of DEPendence and Clinical-Social Fragility instruments for procedures and pathologies, along with the results of the univariate analysis (one-way ANOVA test)

			**Admission**			**Discharge**		
			***N***	**Mean (± SD)**	***P*. value**	***N***	**Mean (± SD)**	***P*. value**
**Conley (fall risk)**	**Procedures**	*Allogeneic HSCT*	55	0.7 (1.5)	** < 0.001**	55	0.9 (1.6)	** < 0.001**
		*Autologous HSCT*	166	1.0 (0.5)		166	1.0 (0.6)	
		*CAR-T therapy*	20	0.9 (0.4)		20	1.6 (0.5)	
	**Pathologies**	ALL	8	2.3 (3.9)	** < 0.001**	8	2.3 (3.3)	** < 0.001**
		AML	20	0.7 (0.7)		20	0.8 (0.7)	
		Ly	86	0.9 (0.6)		86	1.0 (0.9)	
		MDS/MPS	17	0.3 (0.6)		17	0.4 (0.6)	
		MM/PCD	98	1.0 (0.5)		98	1.0 (0.5)	
		Other	12	0.6 (0.8)		12	0.8 (1.0)	
**MEWS (clinical instability)**	**Procedures**	*Allogeneic HSCT*	32	0.9 (0.7)	** < 0.001**	32	0.8 (0.5)	**0.011**
		*Autologous HSCT*	126	0.3 (0.7)		129	0.4 (0.7)	
		*CAR-T therapy*	16	1.1 (1.1)		16	1.1 (0.9)	
	**Pathologies**	ALL	2	1.5 (0.7)	** < 0.001**	3	1.0 (0.0)	** < 0.001**
		AML	10	0.4 (0.5)		10	0.4 (0.5)	
		Ly	67	0.7 (0.9)		68	0.7 (0.9)	
		MDS/MPS	13	0.8 (0.6)		13	0.8 (0.6)	
		MM/PCD	72	0.1 (0.4)		74	0.3 (0.6)	
		Other	10	1.1 (1.2)		9	0.6 (0.5)	
**ICC (caring complexity)**	**Procedures**	*Allogeneic HSCT*	42	25.8 (3.1)	** < 0.001**	42	24.2 (3.2)	** < 0.001**
		*Autologous HSCT*	145	26.2 (1.8)		145	25.8 (2.0)	
		*CAR-T therapy*	18	25.7 (2.7)		18	24.1 (3.8)	
	**Pathologies**	ALL	5	24.0 (4.9)	** < 0.001**	5	22.4 (3.6)	**0.006**
		AML	13	25.2 (3.2)		13	24.2 (2.9)	
		Ly	75	26.2 (1.6)		75	25.7 (2.3)	
		MDS/MPS	16	26.0 (1.9)		16	24.4 (3.7)	
		MM/PCD	86	26.3 (3.2)		86	25.8 (2.1)	
		Other	10	25.3 (2.5)		10	22.7 (3.9)	
**Barthel (functional dependence)**	**Procedures**	*Allogeneic HSCT*	49	93.4 (19.6)	** < 0.001**	49	90.4 (20.1)	** < 0.001**
		*Autologous HSCT*	126	97.3 (10.2)		126	96.8 (10.9)	
		*CAR-T therapy*	17	97.4 (8.7)		17	91.8 (22.8)	
	**Pathologies**	ALL	8	80.0 (30.6)	** < 0.001**	8	80.0 (30.6)	**0.001**
		AML	15	96.7 (12.9)		15	90.0 (17.2)	
		Ly	69	98.0 (6.1)		69	95.9 (13.4)	
		MDS/MPS	16	93.4 (24.9)		16	94.1 (15.1)	
		MM/PCD	75	96.5 (12.7)		75	96.3 (12.8)	
		Other	9	100 (0.0)		9	93.3 (20.0)	
**BRASS (risk of complex discharge)**	**Procedures**	*Allogeneic HSCT*	193	3.2 (2.5)	0.926	193	4.4 (3.1)	**0.005**
		*Autologous HSCT*	206	4.1 (2.4)		206	4.2 (2.6)	
		*CAR-T therapy*	51	4.3 (2.4		51	5.0 (3.4)	
	**Pathologies**	ALL	31	3.7 (3.4)	** < 0.001**	31	3.9 (1.9)	** < 0.001**
		AML	79	3.5 (2.2)		79	4.5 (2.3)	
		Ly	132	3.7 (2.6)		132	4.2 (3.1)	
		MDS/MPS	51	2.8 (1.9)		51	4.2 (3.2)	
		MM/PCD	133	4.4 (2.6)		133	4.5 (2.8)	
		Other	24	3.3 (1.8)		24	4.9 (5.3)	
**Pain**	**Procedures**	*Allogeneic HSCT*	32	0.5 (1.7)	**0.006**	33	0.6 (1.9)	** < 0.001**
		*Autologous HSCT*	83	0.3 (1.1)		84	0.4 (1.2)	
		*CAR-T therapy*	14	0.6 (1.9)		15	0.8 (2.1)	
	**Pathologies**	ALL	4	2.5 (3.3)	** < 0.001**	5	1.4 (3.1)	** < 0.001**
		AML	12	0.0 (0.0)		12	0.0 (0.0)	
		Ly	48	0.5 (1.5)		49	0.7 (1.7)	
		MDS/MPS	12	0.5 (1.5)		12	0.7 (1.5)	
		MM/PCD	44	0.2 (0.9)		45	0.2 (0.9)	
		Other	9	0.1 (0.3)		9	0.9 (2.3)	
**Braden (skin integrity)**	**Procedures**	*Allogeneic HSCT*	51	20.9 (1.9)	0.118	51	20.3 (2.1)	0.877
		*Autologous HSCT*	135	21.4 (2.2)		135	21.3 (2.3)	
		*CAR-T therapy*	18	21.7 (1.5)		18	21.1 (2.3)	
	**Pathologies**	ALL	7	20.3 (1.9)	**0.002**	7	20.6 (1.4)	**0.036**
		AML	19	21.4 (1.9)		19	20.5 (2.6)	
		Ly	74	22.0 (1.5)		74	21.7 (1.9)	
		MDS/MPS	16	20.4 (1.7)		16	19.9 (1.0)	
		MM/PCD	78	21.0 (2.5)		78	20.9 (2.5)	
		Other	10	21.2 (1.7)		10	20.3 (2.7)	
**Sensory perception (subscale of the Braden Scale)**	**Procedures**	Allogeneic HSCT Autologous HSCT CAR-T therapy	51 135 18	3.9 (0.2) 3.9 (0.3) 3.9 (0.2)	**0.004**	51 135 18	3.9 (0.2) 3.9 (0.3) 3.9 (0.2)	0.063
	**Pathologies**	ALL AML Ly MDS/MPS MM/PCD Other	7 19 74 16 78 10	3.8 (0.4) 3.9 (0.2) 3.9 (0.2) 4 (0) 3.9 (3.6) 4 (0)	**0.000**	7 19 74 16 78 10	4 (0) 3.8 (0.4) 3.9 (0.2) 4 (0) 3.9 (0.4) 4 (0)	**0.000**
**Nutrition (subscale of the Braden Scale)**	**Procedures**	Allogeneic HSCT Autologous HSCT CAR-T therapy	51 135 18	3.2 (0.6) 3.5 (0.6) 3.5 (0.5)	0.850	51 135 18	3.0 (0.7) 3.5 (0.6) 3.3 (0.7)	0.538
	**Pathologies**	ALL AML Ly MDS/MPS MM/PCD Other	7 19 74 16 78 10	3.2 (0.5) 3.4 (0.5) 3.5 (0.5) 3.0 (0.6) 3.5 (0.6) 3.2 (0.6)	0.776	7 19 74 16 78 10	3.2 (0.5) 3.1 (0.8) 3.4 (0.6) 2.8 (0.3) 3.5 (0.6) 2.9 (0.9)	**0.018**

*SD* standard deviation, *ALL* acute lymphocytic leukemia, *AML* acute myeloid leukemia, *Ly* lymphoma, *MDS/MPD* myelodysplastic syndromes/myeloproliferative diseases, *MM/PCD* myeloma multiple/plasma-cell disorders, *MEWS* Modified Early Warning Score, *ICC* Index of Caring Complexity, *BRASS* Blaylock Risk Assessment Screening Score

#### CAR-T therapy

CAR-T therapy was used in 10.8% of cases (Table 1), almost exclusively for lymphoma (88.9%). These patients lived locally in 85.2% of cases and had an in-hospital mortality rate of 1.85% (Table 2). The most frequent complications were CRS and neurological toxicities (46.3%), whereas classical transplant complications like GVHD were absent (Table 3). Despite shorter hospital stays than allo-HSCT, CAR-T patients had higher care complexity and risk scores: fall risk (Conley score at discharge 1.6 vs. 1.0 in Auto, 0.9 in Allo, *p* < 0.001), clinical deterioration (MEWS discharge: 1.1 vs. 0.4 and 0.8, *p* = 0.011), and discharge complexity (BRASS score: 5.0 vs. 4.2 and 4.4, *p* = 0.005) (Table 4). Altered mental status at admission was most prevalent in this group (11.11%) (Table 2). Regression analysis confirmed a reduced risk of pressure ulcers (Braden Scale OR 0.292, *p* = 0.047) (Table 5).

**Table 5:** Logistic regression analysis: relationship between the type of treatment, the type of pathologies, the type of complications and the binary outcomes derived from DEP-CSF indices

			**Admission**			**Discharge**		
			**OR**	**(95% CI)**	**P > z**	**OR**	**(95% CI)**	**P > z**
**Conley (fall risk)**	**Procedures**	*Allogeneic HSCT*	1			1		
		*Autologous HSCT*	.693	(.250–1.923)	0.482	.583	(.232–1.462)	0.250
		*CAR-T therapy*	.429	(.048–3.810)	0.448	.309	(.036–2.644)	0.284
	**Pathologies**	*ALL*	1			1		
		*AML*	.333	(.038–2.910)	0.320	.529	(.070–3.978)	0.537
		*Ly*	.146	(.022–.967)	**0.046**	.225	(.037–1.364)	**0.105**
		*MDS/MPS*	.187	(.014–2.467)	0.203	.187	(.014–2.467)	0.203
		*MM/PCD*	.303	(.053–1.730)	0.179	.340	(.060–1.920)	0.222
		*Other*	.6	(.066–5.446)	0.650	.6	(.066–5.446)	0.650
	**DRG**	< *60.000€*						
		≥ *60.000€*	1.181	(.254–5.481)	0.832	1.579	(.429–5.808)	0.492
	**Complications**	*No*	1.305	(.154–11.034)	0.807	1.101	(.131–9.241)	0.929
		*aGVHD*	1			1		
		*VOD*	1.305	(.154–11.034)	0.807	1.101	(.131–9.241)	0.929
		*CRS/neurologic toxicities*	1			.978	(.118–8.111)	0.984
		*Sepsis*	1			1		
**MEWS (clinical instability)**	**Procedures**	*Allogeneic HSCT*	1			1		
		*Autologous HSCT*	.248	(.015–4.076)	0.329	.110	(.014–.844)	**0.034**
		*CAR-T therapy*	7.153	(.679–75.313)	0.101	1		
	**Pathologies**	*ALL*	1			1		
		*AML*	1			1		
		*Ly*	.187	(.027–1.297)	0.090	3.369	(.341–33.195)	0.298
		*MDS/MPS*	1			1		
		*MM/PCD*	1			1		
		*Other*	1			1		
	**DRG**	< *60.000€*						
		≥ *60.000€*	3.590	(.369–3.493)	0.271	4.909	(.470–5.117)	0.183
	**Complications**	*No*	1				1	
		*aGVHD*	1				1	
		*VOD*	8.111	(.731–89.941)	0.088	8.994	1	
		*CRS/neurologic toxicities*	6.952	(.639–75.635)	0.111	7.563	1	
		*Sepsis*	1				1	
**Barthel (functional dependence)**	**Procedures**	*Allogeneic HSCT*	1			1		
		*Autologous HSCT*	.288	(.074–1.123)	0.073	.211	(.065–.683)	**0.009**
		*CAR-T therapy*	.550	(.059–5.074)	0.598	.683	(.130–3.588)	0.653
	**Pathologies**	*ALL*	1			1		
		*AML*	.119	(.009–1.425)	0.093	.256	(.032–2.022)	0.196
		*Ly*	.024	(.002–.280)	**0.003**	.075	(.012–.477)	**0.006**
		*MDS/MPS*	.111	(.009–1.325)	0.082	.238	(.030–1.868)	0.172
		*MM/PCD*	.093	(.016–.540)	**0.008**	.093	(.016–.540)	**0.008**
		*Other*	1			.208	(.016–2.599)	0.223
	**DRG**	< *60.000€*						
		≥ *60.000€*	5.142	(1.196–2.210)	**0.028**	1.006	(3.030–3.339)	**0.000**
	**Complications**	*No*	1			1		
		*aGVHD*	1			1		
		*VOD*	2.870	(.311–26.457)	0.352	1.935	(.216–17.323)	0.555
		*CRS/neurologic toxicities*	1			1		
		*Sepsis*	1			5.807	(.493–68.414)	0.162
**BRASS (risk of complex discharge)**	**Procedures**	*Allogeneic HSCT*	1			1		
		*Autologous HSCT*	.620	(.102–3.756)	0.604	.302	(.080–1.133)	0.076
		*CAR-T therapy*	2.585	(.420–15.899)	0.305	1.739	(.513–5.896)	0.374
	**Pathologies**	*ALL*	1			1		
		*AML*	.384	(.023–6.347)	0.504	.907	(.090–9.153)	0.935
		*Ly*	.697	(.070–6.943)	0.759	1.095	(.125–9.526)	0.934
		*MDS/MPS*	1			1.437	(.141–1.458)	0.759
		*MM/PCD*	.458	(.040–5.218)	0.529	.530	(.052–5.326)	0.590
		*Other*	1			1		
	**DRG**	< *60.000€*						
		≥ *60.000€*	2.412	(.458–1.270)	0.299	5.042	(1.807–1.406)	**0.002**
	**Complications**	*No*	3.510	(.377–32.595)	0.269	4.142	(.815–21.039)	0.086
		*aGVHD*	1			1		
		*VOD*	7.977	(1.384–45.960)	**0.020**	9.942	(2.686–36.795)	**0.001**
		*CRS/neurologic toxicities*	1			4.519	(.885–23.054)	0.070
		*Sepsis*	1			6.214	(.682–56.613)	0.105
**Pain**	**Procedures**	*Allogeneic HSCT*	1			1		
		*Autologous HSCT*	.890	(.215—3.678)	0.873	1.200	(.303–4.739)	0.795
		*CAR-T therapy*	1.611	(.238—10.896)	0.625	1.538	(.229–10.325)	0.657
	**Pathologies**	*ALL*	1			1		
		*AML*	1			1		
		*Ly*	.116	(.013–1.016)	0.052	.666	(.064–6.871)	0.733
		*MDS/MPS*	.090	(.005–1.546)	0.097	.363	(.018–7.294)	0.508
		*MM/PCD*	.073	(.007–.718)	**0.025**	.285	(.023–3.427)	0.323
		*Other*	.125	(.007–2.176)	0.154	1.142	(.077–1.694)	0.923
	**DRG**	< *60.000€*						
		≥ *60.000€*	1.927	(.373–9.937)	0.433	1.621	(.320–8.196)	0.559
	**Complications**	*No*	1			1		
		*aGVHD*	1			1		
		*VOD*	1			1		
		*CRS/neurologic toxicities*	2	(.212–18.848)	0.545	4.166	(.688–25.204)	0.120
		*Sepsis*	5	(.415–60.120)	0.205	1		
**Braden (skin integrity)**	**Procedures**	*Allogeneic HSCT*	1			1		
		*Autologous HSCT*	.384	(.185–.798)	**0.010**	.239	(.104–.548)	**0.001**
		*CAR-T therapy*	.384	(.123–1.193)	0.098	.292	(.087–.981)	**0.047**
	**Pathologies**	*ALL*	1			1		
		*AML*	.285	(.028–2.887)	0.288	.361	(.035–3.702)	0.391
		*Ly*	.166	(.019–1.453)	0.105	.185	(.021–1.619)	0.128
		*MDS/MPS*	1.166	(.088–15.457)	0.907	1		
		*MM/PCD*	.266	(.030–2.325)	0.232	.266	(.030–2.325)	0.232
		*Other*	.388	(.031–4.795)	0.461	.666	(.048–9.189)	0.762
	**DRG**	< *60.000€*						
		≥ *60.000€*	1.756	(.601–51.329)	0.303	1.098	(1.430–8.430)	**0.021**
	**Complications**	*No*	4.096	(.482–34.759)	0.196	1		
		*aGVHD*	1.365	(.121–15.344)	0.801	1		
		*VOD*	1.706	(.322–9.042)	0.530	1.588	(.299–8.420)	0.586
		*CRS/neurologic toxicities*	.853	(.221–3.288)	0.818	.794	(.206–3.062)	0.738
		*Sepsis*	1.365	(.121–15.344)	0.801	1		
**Sensory perception (subscale of the Braden scale)**	**Procedures**	*Allogeneic HSCT*	1			1		
		*Autologous HSCT*	.748	(.132–4.214)	0.742	.615	(.141–2.674)	0.517
		*CAR-T therapy*	1.441	(.122–1.692)	0.771	.941	(.091–9.671)	0.959
	**Pathologies**	*ALL*	1			1		
		*AML*	.333	(.017–6.191)	0.461	4687	(.865–25.370)	0.073
		*Ly*	.253	(.022–2.827)	0.265	1056	(.206–5.406)	0.948
		*MDS/MPS*	1			1		
		*MM/PCD*	.157	(.012–2.002)	0.154	1		
		*Other*	1			1		
	**DRG**	< *60.000€*						
		≥ *60.000€*	1.764	(.200–1.552)	0.609	3.196	(.612–16.680)	0.168
	**Complications**	*No*	7.125	(.687–73.791)	0.100	5.666	(.570–56.306)	0.139
		*aGVHD*	1			17	1.314–219.886)	**0.030**
		*VOD*	7.125	(.687–73.791)	0.100	5.666	(.570–56.306)	0.139
		*CRS/neurologic toxicities*	5.343	(.534–53.467)	0.154	4.25	(.443–40.772)	0.210
		*Sepsis*	1			1		
**Nutrition (subscale of the Braden Scale)**	**Procedures**	*Allogeneic HSCT*	1			1		
		*Autologous HSCT*	.252	(.124–.510)	**0.000**	.151	(.068–.337)	**0.000**
		*CAR-T therapy*	.378	(.124–1.148)	0.086	.267	(.082–.868)	0.028
	**Pathologies**	*ALL*	1			1		
		*AML*	.55	(.084–3.589)	0.532	.866	(.129–5.816)	0.883
		*Ly*	.272	(.049–1.499)	0.135	.321	(.058–1.767)	0.192
		*MDS/MPS*	2.8	(.307–25.524)	0.361	1		
		*MM/PCD*	.293	(.053–1.606)	0.157	.293	(.053–1.606	0.157
		*Other*	.933	(.111–7.819)	0.949	1.6	(.167–15.272)	0.683
	**DRG**	< *60.000€*						
		≥ *60.000€*	1.712	(.636–4.610)	0.287	8.173	(1.827–3.655)	**0.006**
	**Complications**	*No*	6.962	(.828–59.048)	0.075	1		
		*aGVHD*	2.320	(.20–26.069)	0.495	1		
		*VOD*	1.547	(.336–7.118)	0.575	1.379	(.299–6.346)	0.679
		*CRS/neurologic toxicities*	1.450	(.376–5.584)	0.589	1.293	(.336–4.978)	0.708
		*Sepsis*	2.320	(.20–26.069)	0.495	1		

*OR* odds ratio, *CI* confidence interval, *P* > *z P*-value, *ALL* acute lymphocytic leukemia, *AML* acute myeloid leukemia, *Ly* lymphoma, *MDS/MPD* myelodysplastic syndromes/myeloproliferative diseases, *MM/PCD* multiple myeloma/plasma-cell disorders, *HSCT* hematopoietic stem cell transplantation, *DRG* diagnosis related groups, *aGVHD* acute graft-versus-host disease, *VOD* veno-occlusive disease, *CRS* cytokine release syndrome

### Logistic regression analysis of DEP-CSF index variations by treatment type, indications, costs, and complications in HSCT and CAR-T therapy

Table 5 presents the results of the binary logistic regression analysis, examining the relationship between the type of cell therapy, the type of pathologies, the type of complications, and the binary outcomes derived from DEP-CSF indices.

#### Fall risk (Conley Scale)

At admission, patients diagnosed with lymphoma had a significantly lower likelihood of being at risk for falls, with an odds ratio (OR) of 0.146 (95% CI 0.022–0.967; *p* = 0.046).

#### Clinical instability (MEWS)

At discharge, individuals who had undergone autologous HSCT showed a markedly reduced risk of clinical deterioration, with an OR of 0.110 (95% CI 0.014–0.844; *p* = 0.034).

#### Functional dependence (Barthel Index and BRASS)

The Barthel Index revealed notable associations.

At admission, patients with lymphoma had a substantially lower likelihood of being dependent (OR 0.024; 95% CI 0.002–0.280; *p* = 0.003), as did patients with multiple myeloma or plasma-cell disorders (OR 0.093; 95% CI 0.016–0.540; *p* = 0.008).

At discharge, autologous HSCT was associated with reduced dependency (OR 0.211; 95% CI 0.065–0.683; *p* = 0.009), as were lymphoma (OR 0.075; 95% CI 0.012–0.477; *p* = 0.006) and multiple myeloma/plasma-cell disorders (OR 0.093; 95% CI 0.016–0.540; *p* = 0.008).

Regarding the BRASS Index, at discharge, the presence of veno-occlusive disease (VOD) was associated with an increased risk of complex discharge planning (OR 9.942; 95% CI 2.686–36.795; *p* = 0.001).

#### Pain

At admission, multiple myeloma/plasma-cell disorders were associated with a significantly reduced risk of experiencing significant pain (OR 0.073; 95% CI 0.007–0.718; *p* = 0.025).

#### Skin integrity (Braden Scale–total score)

Autologous HSCT was associated with a lower risk of being at risk for pressure sore development at both admission (OR 0.384; 95% CI 0.185–0.798; *p* = 0.010) and discharge (OR 0.239; 95% CI 0.104–0.548; *p* = 0.001).

CAR-T therapy also showed a protective association at discharge (OR 0.292; 95% CI 0.087–0.981; *p *= 0.047).

#### Sensory perception (Braden Subscale)

At discharge, the presence of acute graft-versus-host disease (aGVHD) was significantly associated with an increased risk on the sensory perception subscale (OR 17; 95% CI 1.314–219.886; *p* = 0.030).

#### Nutrition (Braden Subscale)

Autologous HSCT was strongly associated with a lower risk of poor nutritional status, both at admission (OR 0.252; 95% CI 0.124–0.510; *p *< 0.001) and at discharge (OR 0.151; 95% CI 0.068–0.337; *p* < 0.001).

CAR-T therapy was also protective at discharge (OR 0.267; 95% CI 0.082–0.868; *p* = 0.028).

#### Healthcare resource utilization (DRG ≥ 60,000€)

At admission, patients with functional dependence were more likely to incur DRG-related costs ≥ 60,000€, with an OR of 5.142 (95% CI 1.196–22.210; *p* = 0.028).

## Discussion

To the best of our knowledge, this is the first study to investigate DEP-CSF indices in the context of hematopoietic stem cell transplantation (HSCT) and chimeric antigen receptor T-cell (CAR-T) therapy. Extreme DEP-CSF indices, either elevated or reduced, were predominantly observed in patients receiving CAR-T therapy—both at admission and discharge—potentially reflecting increased risks of falls, clinical deterioration, and greater care complexity. In contrast, patients undergoing allogeneic HSCT exhibited lower levels of functional independence. Although autologous HSCT was the most frequently performed treatment in this sample, no associations with extreme DEP-CSF indices scores were observed.

Part of the study period coincided with the SARS-CoV-2 pandemic. While CAR-T technology continued to expand during this time [25, 26], the pandemic may have influenced patient selection, access to care, and hospitalization criteria. However, since DEP-CSF data were routinely collected through electronic nursing documentation, no major gaps in data collection were identified. Regarding risk prevention, falls are a relatively rare event in these patients [27]. In our study, although patients undergoing CAR-T had a higher mean Conley score compared to patients undergoing allogeneic or autologous HSCT procedures, this score was < 2 (cutoff for patients at risk). Moreover, the mean score, while raised at discharge, was also < 2. Logistic regression confirmed that patients diagnosed with lymphoma had a significantly lower likelihood of being at risk for falls.

Regarding clinical deterioration, CRS and ICANS are the most common complications related to CAR-T treatment [28, 29], and while they require specific, validated grading systems [30–32], our study explored the potential role of MEWS as a general clinical deterioration marker. Although MEWS is not specific for inflammatory or neurotoxic syndromes, it may help flag early signs of instability during hospitalization. However, its limited sensitivity to symptoms like isolated fever or neurologic changes should be acknowledged, and its value in predicting or tracking CRS/ICANS episodes remains to be validated. The mean MEWS scores emerging from this study were particularly low, indicating a low risk of clinical deterioration. This may be related to the time points at which they were evaluated (at admission and discharge). Logistic regression results revealed that auto-HSCT patients exhibited a markedly reduced risk of clinical deterioration at discharge. A longitudinal assessment of MEWS trajectories during hospitalization and their correlation with clinical outcomes could offer valuable prognostic insights and represents a compelling direction for future research.

Both CRS and neurotoxicity are important recognized complications for patients undergoing CAR-T therapy [29]. Use of the BRASS discharge complexity scale, as in this study, may facilitate identification of the need for post-discharge monitoring and caregiver education to manage symptoms. Altered mental status at admission was significantly more common in CAR-T therapy patients compared to allogeneic and autologous HSCT patients. However, despite a higher frequency of altered mental status at admission among CAR-T patients, mental status at discharge was not significantly worse compared to the other groups. While the literature highlights the importance of monitoring cognitive function and identifying individuals at risk for ICANS [30], potential factors influencing mental status at the point of admission should also be investigated further. Patients undergoing CAR-T therapy also demonstrated lower risks on the Braden scale’s sensory perception subscale at discharge [33].

Complications of HSCT often involve pain (e.g., mucositis) [3], so the low levels of pain across all three treatments in this sample warrant prospective investigation. This finding was somewhat unexpected, particularly among multiple myeloma patients, for whom bone pain and neuropathy are common. One possible explanation may lie in pain control strategies or underreporting, but our dataset did not include information on comorbidities or analgesic regimens, limiting interpretation. Future studies should consider collecting data on supportive care and symptom management to better understand the pain experience in this population. Logistic regression revealed that patients with multiple myeloma or plasma-cell disorders were significantly less likely to experience severe pain at admission, although pain is known to be frequent among patients with myeloma [34, 35].

A higher in-hospital mortality rate was observed in patients undergoing allogeneic HSCT, consistent with findings from the literature. In our study, causes of mortality were not examined or corrected for the hematopoietic cell transplantation-specific comorbidity index. The complications of allogeneic HSCT and its longer hospitalizations [36] may explain the lower functional independence index (Barthel) observed among these patients, especially at discharge. Regression analysis showed that autologous HSCT was associated with a reduced risk of dependency at discharge. Moreover, patients with lymphoma and multiple myeloma also showed lower dependency risks, which may reflect both disease characteristics and the fact that the majority of these patients in our cohort received autologous transplants.

While the alignment between specific diagnoses and treatment types (e.g., ALL and CAR-T or allo-HSCT) is expected, it is important to describe these distributions to contextualize the differences observed in functional dependence, care complexity, and risk profiles. Hematological indications revealed that patients with ALL were at greater risk for falls, clinical deterioration, car complexity, and lower functional independence compared to other neoplasms. These findings must be cautiously interpreted due to the small sample size (6.4%) and the lack of adjustments for procedures received. The majority of ALL patients underwent allogeneic HSCT or CAR-T therapy, treatments associated with higher DEP-CSF indices and lower functional independence. Additionally, patients with MDS/MPS exhibited an increased risk of pressure sores at discharge [37–43].

Finally, patients with MM and PCD displayed higher BRASS scores at discharge. This aligns with reports that MM patients experience a high symptom burden, with moderate-to-severe impairments in well-being, fatigue, pain, and drowsiness [44]. This highlights the importance of a tailored discharge plan to address specific needs in this population, particularly regarding post-discharge care and follow-up.

From a policy perspective, the study underscores the importance of integrating DEP-CSF indices into patient assessments, as they can highlight active problems or risks, addressing both physical (e.g., functional dependence, pressure injuries) and social aspects (e.g., post-discharge challenges). The collective use of DEP-CSF indices, rather than isolated administration of independent tools, reflects current clinical practice, where these indices are routinely assessed by trained nursing staff. This integrated model supports a multidimensional understanding of patient fragility and care complexity without increasing the burden on patients. While the operational workload lies primarily with nursing staff, in our institution, these assessments are part of routine clinical documentation and do not represent an additional burden. Moreover, the model aligns with quality standards required by JACIE (Joint Accreditation Committee of ISCT and EBMT) and JCI (Joint Commission International), both of which mandate structured evaluation of patient-related risk factors as part of standard care in transplant and CAR-T programs. Therefore, DEP-CSF is potentially generalizable to similarly accredited centers [45, 46].

While this study was conducted in an inpatient setting, the potential application of DEP-CSF indices in outpatient care—such as pre-admission assessment or post-discharge follow-up—warrants further investigation. In ambulatory contexts, these tools could support early identification of emerging risks, help plan transitions of care, and enhance monitoring of patients receiving cell therapy in day-hospital or home-based programs. The indices, such as Barthel, Braden, and Conley, align with the cognitive/perceptive model of the Professional Assessment Instrument used in some Italian hospitals [21]. Including these indices in a Nursing Minimum Data Set could streamline the systematic recording of standardized nursing data and improve the quality of care [47].

On a macro level, policymakers should develop strategies tailored to the complex needs of HSCT and CAR-T patients, focusing on mitigating risks and improving outcomes through standardized guidelines. At the meso level, healthcare organizations should adopt DEP-CSF indices in clinical routines, train multidisciplinary teams, and implement advanced patient monitoring systems. On a micro level, healthcare professionals should emphasize holistic care, improve communication with patients and caregivers, and enhance post-discharge support.

While innovative, this study has several limitations. Its descriptive design precludes any causal inferences. Data collection limited to admission and discharge may not fully capture the clinical trajectory or fluctuations in patients’ conditions during hospitalization. The indices examined were not correlated with specific outcomes (e.g., falls, complications, or readmissions) nor assessed at multiple intermediate time points. Furthermore, potential confounding factors, such as variations in treatment protocols or supportive care measures, were not controlled for, and hematological diagnoses were not included in the adjustment. These factors may limit the generalizability and applicability of the findings. Future research should aim to explore the predictive validity of these indices, evaluate their association with clinically relevant outcomes, and incorporate longitudinal assessments throughout hospitalization.

In summary, assessing both dependency and clinical-social fragility indices may provide useful information for identifying patients potentially at higher risk during hematopoietic stem cell transplantation (HSCT) and CAR-T cell therapy. These indices offer an overview of a patient’s functional status and social support context, which could inform individualized care planning. However, given the study’s limitations, these findings should be interpreted with caution, and further research is required before these indices can be integrated into routine clinical decision-making or resource allocation strategies.

## Data Availability

No datasets were generated or analysed during the current study.
